# Development of a Low-Cost Electronic Nose with an Open Sensor Chamber: Application to Detection of *Ciboria batschiana*

**DOI:** 10.3390/s23020627

**Published:** 2023-01-05

**Authors:** Piotr Borowik, Tomasz Grzywacz, Rafał Tarakowski, Miłosz Tkaczyk, Sławomir Ślusarski, Valentyna Dyshko, Tomasz Oszako

**Affiliations:** 1 Faculty of Physics, Warsaw University of Technology, ul. Koszykowa 75, 00-662 Warszawa, Poland; 2 Institute of Theory of Electrical Engineering, Measurement and Information Systems, Faculty of Electrical Engineering, Warsaw University of Technology, ul. Koszykowa 75, 00-662 Warszawa, Poland; 3 Forest Protection Department, Forest Research Institute, ul. Braci Leśnej 3, 05-090 Sękocin Stary, Poland; 4 Ukrainian Research Institute of Forestry and Forest Melioration Named after G. M. Vysotsky, 61024 Kharkiv, Ukraine

**Keywords:** electronic nose, gas sensor chamber, air flow modeling, odor detection

## Abstract

In the construction of electronic nose devices, two groups of measurement setups could be distinguished when we take into account the design of electronic nose chambers. The simpler one consists of placing the sensors directly in the environment of the measured gas, which has an important advantage, in that the composition of the gas is not changed as the gas is not diluted. However, that has an important drawback in that it is difficult to clean sensors between measurement cycles. The second, more advanced construction, contains a pneumatic system transporting the gas inside a specially designed sensor chamber. A new design of an electronic nose gas sensor chamber is proposed, which consists of a sensor chamber with a sliding chamber shutter, equipped with a simple pneumatic system for cleaning the air. The proposal combines the advantages of both approaches to the sensor chamber designs. The sensors can be effectively cleared by the flow of clean air, while the measurements are performed in the open state when the sensors are directly exposed to the measured gas. Airflow simulations were performed to confirm the efficiency of clean air transport used for sensors’ cleaning. The demonstrated electronic nose applies eight Figaro Co. MOS TGS series sensors, in which a transient response caused by a change of the exposition to measured gas, and change of heater voltage, was collected. The new electronic nose was tested as applied to the differentiation between the samples of *Ciboria batschiana* fungi, which is one of the most harmful pathogens of stored acorns. The samples with various coverage, thus various concentrations of the studied odor, were measured. The tested device demonstrated low noise and a good level of repetition of the measurements, with stable results during several hours of repetitive measurements during an experiment lasting five consecutive days. The obtained data allowed complete differentiation between healthy and infected samples.

## 1. Introduction

Electronic nose devices [1,2,3] apply an array of nonspecific gas sensors with an overlapping scope of gas detection. The collected pattern of sensors’ response to the presence of measured odors does not allow for analyzing the chemical composition of analyzed samples and relies on machine learning pattern recognition algorithms for the classification of samples or determination of the smell intensity.

Sample handling [4] in the electronic nose measurement is an active subject of research, containing the preparation of gas mixtures and the design of a dedicated measurement setup. An important component of an electronic nose device is a sensor chamber [5], where gas sensors are mounted and usually to which a sample of measured gas is transported by a dedicated pneumatic system. In many reported electronic noses constructions, the chamber is just a box containing sensors, but there are also more sophisticated solutions designed with the help of fluid dynamics simulations. However, also a class of simple devices, or measurement setups, can be noticed, which we can call open sensor chambers. In these devices, sensors are placed close to the odor source without any pneumatic system transporting the measured gas. To acknowledge other research in the field of electronic nose sensor chamber construction, we provide a short review of this field in Section 2.

The present paper proposes another approach to the gas sensor chamber design of a low-cost electronic nose, in which a measured sample of the gas is not transported to the sensors’ area. Still, a simple pneumatic system is added to provide the clean air used for efficient determination of the sensors’ baseline response level and sensors cleaning after measurement.

The new electronic nose device was applied to differentiate between odors emitted by healthy and infected samples of oak acorn pathogenic fungi. We would like to acknowledge here the other research on electronic nose applications in forestry and agriculture dealing with similar types of applications. There are some research papers focused on the detection of fungal pathogens in stored seeds or grains. The studies of samples of cereal [6,7], rice [8,9], rapeseed [10,11], and wheat [12] were reported. The identification of fungal species by electronic noses was reviewed by Mota et al. [13]. Several reviews of the applications of electronic noses, focusing on forestry and agriculture [5,14,15,16,17,18], are available.

Storage of oak acorns is extremely important for maintaining production continuity in forest nurseries. This tree species produce new acorns at long intervals, sometimes for up to eight years [19]. On the other hand, it is the third most common forest-forming tree species in Polish nurseries. Therefore, it is necessary to store the acorns in special storerooms. In most cases, acorns are collected in barrels in the years of sowing, where they are stored for the following years. Unfortunately, if infected acorns are found in such conditions, the infection can easily spread to neighboring nuts. Spoiled acorns can be detected by visual inspection, which can be automated using computer vision methods [20]. Of particular importance for acorn storage is the detection of the fungus *Ciboria batschiana*, which causes a disease known as acorn mummification. This fungus infects acorns before the fall harvest, and if they are not cleared of the fungus by thermotherapy, they may suffer severe losses during subsequent storage. However, since thermotherapy is time-consuming, expensive, and sometimes has undesirable side effects, its use is not justified unless it is known whether and to what extent the seedling is infected [21]. Because of this, it seems justified to use an electronic nose for rapid and non-invasive detection of the fungus *Ciboria batschiana* in the collected acorns.

The goal of the manuscript is to demonstrate the new construction of a low-cost electronic nose, in particular, the new proposal for the sensor chamber. The advantage of the new chamber is the ability to perform measurements of gas samples of small volume, without the transport of the sample by a pneumatic system, and thus without dilution of the sample by a carrier gas. On the other hand, the construction allows efficient sensor cleaning between measurement cycles of various samples. Samples of agar medium and *Ciboria batschiana* of various coverage of Petri dishes, thus various odor intensities, were used in a test-bed of experimental measurements.

The paper is organized as follows. In Section 2, we review various constructions of electronic noses sensor’s chambers designs and reported attempts of the airflow simulations in that component of devices. In Section 3, we describe our construction of an electronic nose, especially the new design of the sensor chamber, which is described in Section 3.2. In Section 4, we describe the preparation of samples used in our experiments and the measurement procedure. In Section 5, we report the results of the performed measurements and performed an analysis of the collected data. Discussion of the results is provided in Section 6. We summarize our research in Section 7.

## 2. Electronic Noses Sensor Chamber Designs

### 2.1. Sensor Chambers with Pneumatic Gas Supply

Most of the electronic nose constructions reported in research papers contain a pneumatic system of gas supply, which helps to automatize the measurement process and to maintain repeatable conditions of measurement cycles. In many of the reports, the sensor chamber is designed as a container without any special design of its shape. However, some research proposed more sophisticated constructions, some of which we would like to review here.

Daqi et al. [22] demonstrated a circular shape of the gas sensor chamber, in which the flow of the gas flows in a channel, sequentially from one sensor to the next one. Sequential placement of sensors, but in the linear-shaped sensor chamber, was examined by Bakar et al. [23] and found inferior to the case of sensor placements in a wider cylindrical sensor chamber. Di Francesco et al. [24] proposed a radially symmetric measurement chamber with a radially symmetric flow splitter, which helps to achieve homogeneous flow conditions and avoids the appearance of stagnant regions and recirculating zones. In addition, in the proposed construction, the gas paths from the inlet to the sensors and from the sensors to the outlet have the same length, which permits the achievement of controlled and repeatable gas composition for all sensors. That allows us to achieve measurements in the same physical conditions. Wu et al. [25] proposed a cylindrical shape of the sensor chamber with a centrally located air outlet and an air inlet on the side of the chamber, optimized to reduce turbulent regions of the airflow. Bionic sensor chambers inspired by mammal nasal cavities were proposed by several researchers [26,27]. A recent review by Cheng et al. [5] of various approaches to sensor chamber designs is available.

### 2.2. Sensor Chambers without Pneumatic Gas Supply

There are some research papers where the authors presented the development of electronic noses in which measured gas is not supplied to the gas sensors by a dedicated pneumatic system. In this section, we would like to review some of such approaches, since they have some common features with the open sensor chamber we used in the present work.

Li et al. [28] proposed a headspace-integrated nose and its application in the discrimination of Chinese medical herbs. It consists of a measurement bottle in which the sample is placed, and odor is diffused towards the gas sensors embedded in a plastic cap of the sample bottle. Wu et al. [29] presented an electronic nose dedicated to the monitoring of flammable liquids, with a stainless steel air bottle and sensor chamber integrated inside. The samples of liquids were heated, and evaporated volatiles were transported to the vicinity of the sensors with the help of a fan. Kuchmenko et al. [30] presented an application for an electronic nose device equipped with piezoelectric sensors. The sensors were located inside a cylindrical sensor chamber, not equipped with any airflow installation, but just placed close to the measured sample. The portable electronic nose was applied to analyze the smell of nasal secretions in calves with the goal of a noninvasive diagnosis of infectious bronchopneumonia. Yavuzer [31] presented studies of the application of a low-cost electronic nose to the determination of fish quality parameters. A plastic food storage box with a lid, cut to pass the sensor heads from the sides, was used as a sensor chamber of its device. A piece of fish meat sample was placed in the box, and the measurements were made by closing the lid.

The previously reported low-cost electronic noses built in our laboratory [32,33,34] were constructed as a round probe, fit to the size of a Petri dish or jar opening hole, and manually moved from the clean air conditions to the measured sample. The noses were used to classify samples of pathogenic oomycetes *Phytophthora plurivora, Pythium intermedium* and fungi *Fusarium oxysporum, Rhizoctonia solani*. A similar method of operation of an electronic nose probe was reported by Fuentes et al. [35] for early detection of aphid infestation and assessment of insect–plant interaction in wheat. Moreover, an electronic nose reported by Gonzalez Viejo et al. [36], used to assess beer quality, had a form of a manually moved probe placed directly on the top of a glass.

### 2.3. Modeling of Sensor Chambers

A goal of optimization of the sensor chamber design is an effective exposure of the sensors’ measured gases. The sensor chamber should be designed in such a way that the flow of the gas is homogeneous, all sensors are exposed to the same odor concentration, and the provided gas composition is controlled, allowing the maintenance of the constant gas composition during sensors’ exposure and abrupt change of the gas from reference conditions to measured gas and vice versa [4,37].

Falcitelli et al. [38] reported a 3D fluid dynamics simulation of a sensor chamber with the goal of ensuring that the transient time necessary to reach uniform and stationary conditions of gas distribution in the chamber is much shorter than the sensor response time. An optimized chamber was proposed by modifications of inlet and outlet profiles and the introduction of diffusers, allowing one to break up the jet and increasing the uniformity of the flow. Scott et al. [39] demonstrated flow dynamic simulations of a series of improvements and optimizations of the sensor chamber structure. Di Francesco et al. [24] optimized its sensor chamber construction using simple one-dimensional models under isothermal conditions, which was possible due to the geometric simplicity of the device. Viccione et al. [40,41] used fluid dynamic modeling to examine the effects of the diffuser employed in the sensor chamber. In its research, volatile discharge was examined as a function of both geometries and injected flow rate. Their studies were performed using a 3D model of the sensor chamber and numerically solving the Navier-Stokes transport equations. Wu et al. [25] presented a low-cost portable electronic nose for cigarette brand identification. They used computational fluid dynamics simulations to optimize the sensor chamber structure and ensure the uniformity of airflow distribution. Jin et al. [42] and Wang et al. [27] studied a series of bionic chambers of electronic noses inspired by mammalian nasal structure. The chambers were optimized using air flow simulations, and several baffles were added to direct gas flow. Another inspiration of nasal structure, for the construction of a bionic sensor chamber, was reported by Villarreal et al. [26] and Chang et al. [43], in which the effective transport of odor to sensors and exhalation of gas from the chamber was achieved. Dohare et al. [44] reported a fluid dynamics simulation of cuboidal electronic nose sensor chambers with three different three-dimensional models with single and multiple inlets and outlets. Furthermore, they studied the influence of the introduction of four baffles directing the flow of the air in the chamber. Zou et al. [45] used computational flow dynamics methods to improve the sensor chamber structure by adding plates helping to stabilize the gas flow. He also demonstrated a reduction of the sensor response noise in the optimized structure.

## 3. Electronic Nose Device Used in the Experiments

### 3.1. Sensors and Electrical Circuit

**Figure 1:**
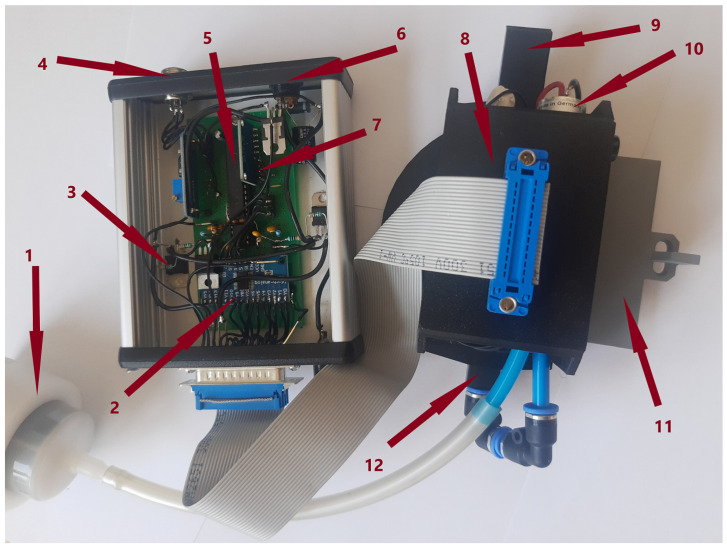
Photo of the electronic nose electrical device. The indicated components are as follows: 1—charcoal filter, 2—16-channel multiplexer CD74HC4067M, 3—heaters voltage stabilizer, 4—USB-cable connector, 5—ATMEGA processor unit, 6—power supply connector, 7—ADS 1115 analog-digital converter, 8—the block of sensors’ probe, 9—gas exhaust, 10—pump, 11—sensor chamber shutter, and 12—ambient gas inlet.

The electrical circuit and sensors used in our electronic nose device are basically the same as it was used in the previous construction for our team [34]. For the reader’s convenience, we would like to provide here a short summary of it. The nose consists of two main parts: the sensors probe, in this case, sensors mounted in the sensor chamber, and the main electronic unit connected to the computer. We used various types of metal oxide sensors manufactured by Figaro Co., Japan, a list of which one can find in Table A1 in Appendix A. The device uses the ATmega 328P-PU microcontroller, which controls all the communication between sensors and the computer. The measurements or sensors’ resistance are read using a multiplexer one by one with a delay of a few milliseconds, so we can assume that they represent the sensors’ conditions of the same moment. The sensor read cycles are repeated each 0.75 s. All readings are sent to a computer and archived in a text file. The electrical circuit of our electronic nose can modulate the sensors’ heater voltage, which leads to the modulation of the operation temperature of the sensors. The changes could be made in every single sensor reading cycle from 0 to 7 V with a step of 30 mV. We modulate the voltage supplied to the sensor heater in the same manner for all sensors mounted in the device [34]. That allows for keeping the construction of the electronic nose simple and low-cost. Such an approach does not mean that the internal temperature of all sensors is the same, as it depends on their resistance and heat generation in heaters. The main unit has wires to connect with the sensors, a USB cable to connect to the computer, and a 12 V DC power supply. The supplied voltage is divided into two separate sub-circuits. The first one is stabilized at the level of 5 V, which supplies most of the electronic parts and the sensors’ readings. The other voltage sub-circuit is the adjustable voltage that powers up the sensors’ heaters. A photo of the electronic nose device, with marked components, is presented in Figure 1.

### 3.2. Sensor Chamber Structure and Mode of Operation

**Figure 2:**
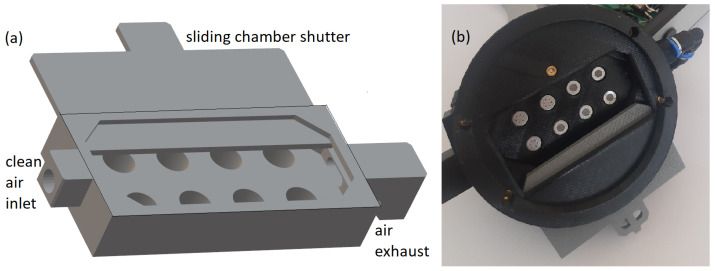
Electronic nose sensor chamber: (**a**) schematic driving, and (**b**) real photo. The view from the bottom when the chamber shutter is open.

In Figure 2a, we present a schematic driving of the sensor chamber designed for the proposed construction of the electronic nose. It can be described as a block of material with hollow holes for the sensors. A sliding shutter closes the chamber, leaving a small empty space inside, between the sensors and the shutter. In the schematic driving of Figure 2a, on the left side, an air inlet is located, and on the right side, the air outlet. In the assembled device, the air inlet is connected by a pipe to a pneumatic pump, which forces the airflow inside the chamber when the shutter is closed. The air outlet directs the flowing air so as not to blow toward the measured sample.

In the closed state, the sensor chamber may be brought close to the sample to be measured, such that a shutter separates the sensors from the gas sample to be measured. At this time, the pump is turned on, forcing the airflow inside the chamber, thanks to which it is possible to determine the baseline state of the measured voltage determining the resistance of each sensor.

It is worth emphasizing here that the air is pumped into the chamber by the pump, and not sucked in, as is the case in other electronic nose designs. Such a change in the direction of the pumped air has an important purpose. The device contains moving parts and is not completely sealed. When the pump is activated, air can penetrate through the gaps between the shutter and the walls of the chamber. When the clean air is pumped into the chamber, it can escape through these leaks, but air from the outside, potentially containing pollutants with odor, is not sucked in. The air entering the pump passes through a carbon filter that allows it to be cleaned.

When the shutter is opened, the pump is turned off, and the airflow stops. Opening the shutter causes the sensors to obtain air from the outside, which contains the odor that we would like to detect. In our experiments, the electronic nose sensor chamber was applied to the Petri dish containing the measured sample. For that reason, the prototype of the constructed device (Figure 2b) is designed to fit the Petri dish.

The dishes with samples were kept closed between measurements, and in their volume, the odor is concentrated. Since the volume of the air contained in the sensor chamber is small, the gas under-measure is only slightly diluted. That is an important difference when we compare to other common constructions of electronic noses in which the air is sucked by the pneumatic pump and a measurement lasting a few minutes requires a substantial volume of transferred gas, which is diluted by clean air. Moreover, the proposed design of the sensor chamber and the mode of operation allows us to keep constant gas concentration during the whole measurement cycle. That is much more difficult to achieve with the standard sensor chambers with a pneumatic gas supply. When the measurement cycle lasts a few minutes, and only a small sample of measure odor is available, sucking the gas and mixing it with air from the environment usually leads to a significant decrease in odor concentration after a short time.

In the next stage of the measurement procedure, the shutter is closed, and the pump is turned on, leading to the cleaning of the sensors. Moreover, in this stage, the sensor’s response may be collected, as it may contain useful information, allowing one to differentiate between various samples’ odor categories. A more detailed discussion of the data collected during the measurement process is provided in Section 3.4.

### 3.3. Airflow in the Proposed Sensor Chamber

In the research reviewed in Section 2.3, the goal of airflow modeling consisted of the optimization of the sensor chamber for working conditions when the measured gas was transported to sensors. In the proposed construction, the role of airflow is different, as it is used only to establish repeatable conditions of baseline level measurement and to allow sensor cleaning. However, the requirements for these are similar, so as to avoid stagnant regions, in which clean air is not efficiently transported, and either sensors are not cleared efficiently, or cleaning takes a much longer time. Efficient cleaning should be guaranteed for all sensors in the chamber. Laminar flow conditions are also expected, as this helps to achieve the cleaning of the sensor chamber without vortices, which could lead to the push back of the odor inside the chamber and degrade cleaning efficacy.

To verify if the proposed construction efficient cleaning of the sensor area, we performed the airflow simulations using COMSOL Multiphysics 5.2.0.166 (COMSOL Inc., Burlington, NJ, USA). Navier-Stokes equations were solved assuming an incompressible flow with no-slip conditions on the walls. Expected conditions of a pressure of 1 atmosphere, a temperature of 300 K, and an average velocity at the chamber inlet of 0.2 m/s were used.

**Figure 3:**
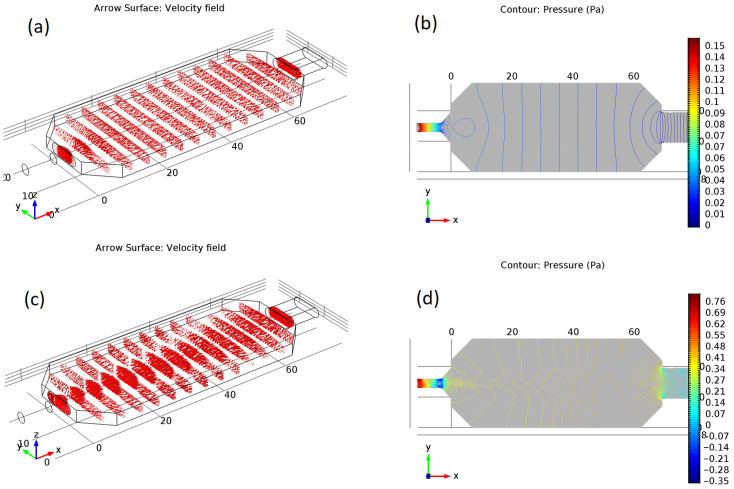
Distribution of air velocity (**a**,**c**) and pressure (**b**,**d**) in sensor chamber in closed state, obtained by airflow simulations using COMSOL Multiphysics package. Air input velocity was set to (**a**,**b**) 0.2 m/s and (**c**,**d**) 2 m/s; temperature 300 K.

In Figure 3, we present the results of the performed airflow simulations of the chamber structure in the state with a closed shutter, as such configuration is used when the airflow is forced by the pump. We chose the initial conditions of the expected flow velocity of 0.2 m/s. The simulations of the airflow in such conditions are presented in subfigures (a) and (b).

As one can notice in Figure 3a, the distribution of air velocity throughout the interior of the chamber array is uniform. Moreover, Figure 3b of the air pressure distribution exhibits constant pressure lines that are straight and perpendicular to the walls of the chamber. That assures that the goal of the efficient sensor chamber cleaning is fulfilled.

In Figure 3c,d, we present results of airflow simulations for much higher velocities of the order of at least 2 m/s. We can observe the appearance of air vortices when the air can be pushed back. Moreover, another drawback of the high air velocity conditions that can be observed in these figures is that sensors located in various positions in the chamber experience different conditions of pressure and flowing gas. Moreover, such conditions are much more distant from the stable ones for the case without maintaining airflow. That can lead to increased noise and instability of measured results.

It is difficult to experimentally determine airflow distribution in a sensor chamber. One of the methods could be the usage of a transparent chamber and visualizing air streams, as in colored smoke in a wind tunnel [46]. However, such an approach can also be limited. In our research, we applied an indirect verification of laminar conditions of gas flow in the chamber. We prepared an experimental setup in which between the air pump and sensor chamber we mounted a flow meter. We were able to set the air pressure generated by the pump by modification of the pump supply voltage. We collect characteristics of the air velocity versus voltage. We found a linear character of the curves, without any noticeable deviations, which supports the expectation, that in the ranges of used input airflow velocity, we maintain the laminar airflow conditions inside the chamber.

### 3.4. Mode of Electronic Nose Operation—Collected Signals

In Section 3.2, we already described the mode of operation of the electronic nose sensor chamber. Presently, we would like to give more details concerning the signal collected from the sensors during one cycle of measurement and data preprocessing.

**Figure 4:**
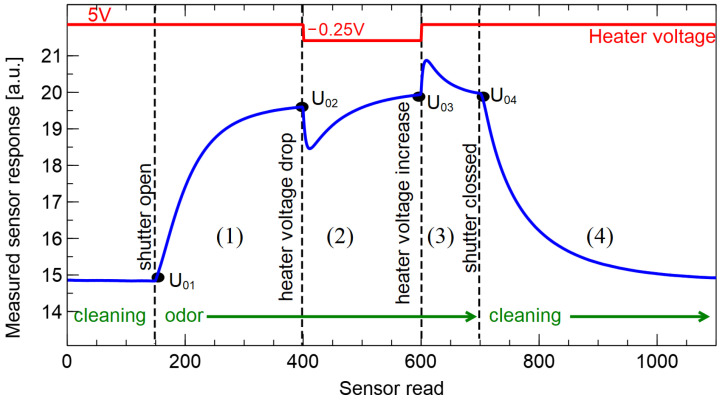
Schematic visualization of the mode of electronic nose operation during one measurement cycle. The measured sensor response is expressed as arbitrary units, as it is the raw output from ADC converter of voltage read on a resistor [32,33,34,48]. Characteristic moments of measurement procedure as opening/closing of the sensor chamber shutter and heater voltage modulation moments are indicated by vertical lines. Four stages of signal observation time (1)–(4), and characteristic baseline voltages of these stages U_01_–U_04_ are marked. The X-axis represents consecutive reads of sensor response magnitude, every 0.75 s. In the example, a response of the TGS 2620 sensor is plotted.

The measured response of the MOX types of sensors is the change of their resistance, which changes in the presence of the gases [47]. There are several types of electronic circuit topologies commonly used for the capture of the MOX sensors’ response [48]. In our case, we measure the voltage on a resistor serially connected to the sensor [32,33,34]. In Figure 4, we present an example of a typical shape of measured response from one of the sensors. A measurement cycle starts from the collection of the voltage measured in clean air conditions. In our experiment, that period lasted about 2 min and we used the output in this region to assure that the response has a flat characteristic, meaning that sensors are sufficiently cleaned and in a stable condition.

As presented in Figure 4, using the device that we describe, we collected transient characteristics of the sensor’s response in four observation stages (1st) after the change of exposure sensors exposure from clean air to measured gas, which is started by the chamber shutter opening; (2nd) after a drop of sensor heater voltage, when sensors are exposed to measured gas; (3rd) after an increase in sensor heater voltage; and (4th) relaxation and cleaning of sensors after chamber shutter closing and turning on the air pump, which changes the sensors’ exposure conditions from measured gas to clean air.

At the moment of starting each observation time, a magnitude of the sensor response is used to normalize the response characteristics in that stage of observation [34]. Such responses can be used to extract the features that are used for training classification models used for differentiation between studied sample categories.

In the presented research, we applied a rectangular profile of the sensor heater modulation, with a relatively shallow drop in the heater voltage, close to the voltage recommended by the manufacturer. In our previous research [34], we verified that a shallow modulation of the range of −0.3 V allows to the collection of data necessary for differentiation between various samples of fungi odors. We compared various magnitudes of modulations and concluded that an increase in the depth of modulation does not provide additional information for differentiation between samples. In the current research, we slightly reduced the depth of the heater modulation voltage to −0.25 V. We decided to use only a single step of sensor heater modulation, as that allows us to reduce the measurement time.

## 4. Measured Samples

### 4.1. Samples’ Preparation

**Figure 5:**
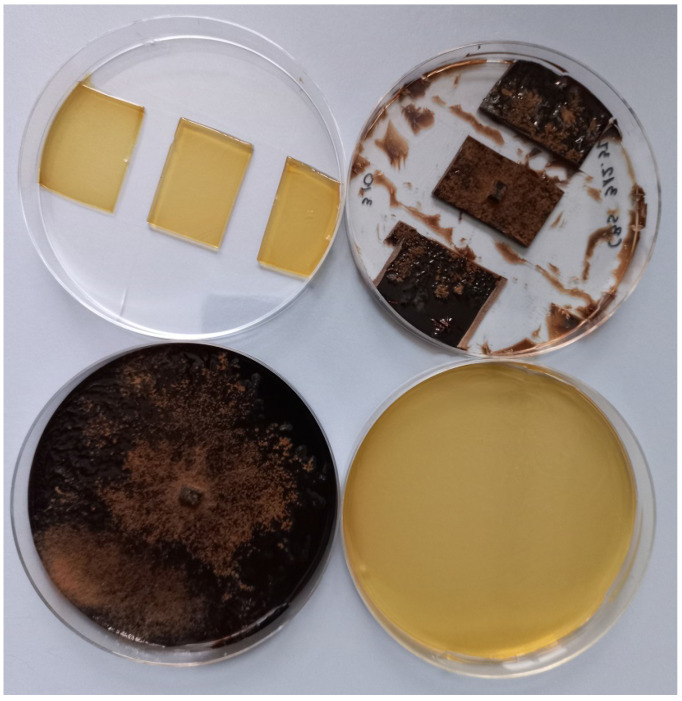
Exemplary photo of the experimental variants of Petri dishes containing measured samples. *Ciboria*—brown, Medium—yellow.

The *Ciboria batschiana* fungal strains used for this study were purchased from the Westerdijk Institute (The Netherlands) and then distributed into new 9 cm diameter Petri dishes containing malt extract agar—MEA (30 g/L malt extract, 5 g/L mycological peptones, 15 g/L agar). The dishes were incubated for five weeks at about 24 °C in daylight. After five weeks, the mycelium had grown on the entire plate. From the prepared dishes fully covered with *Ciboria batschiana* mycelium, the following experimental variants were isolated: two dishes fully covered with the fungus, two dishes partially covered with the fungus (about 1/3 of the plate surface), two dishes with only the MEA medium and two dishes partially covered with the medium only (about 1/3 of the pan surface). For variants where there was less than the whole dish, the excess medium was removed with a sterile scalpel (Figure 5).

### 4.2. Measurement Procedure

The odor measurement procedure was performed in the same way as in previous works [32,33,34]. This has been covered in detail in previous articles, so we only cover the most important parts here. Two complete series of measurements were performed for all samples on each day. The sample sequence was selected using a random number generator at the beginning of each series.

At the beginning of each measurement, the sensor chamber (with the shutter closed) was placed on an open Petri dish. Each measurement included 1200 sensor readings; readings were taken approximately one second each. The first 150 readings formed the baseline. Then, the chamber shutter was opened manually. After 550 readings, the chamber shutter was closed, thus completing the process of recording volatile odorous compounds. The last 500 resistance readings recorded the sensors’ relaxation and cleaning. When not measured, all samples in Petri dishes were covered to avoid contamination. All measurements were made in a laminar flow booth (Telstar, Bio II Advance) at 21 °C with the air supply on. This made it possible to maintain the controlled temperature and humidity conditions throughout the experiment.

Measurements were made continuously over five days (14–18 November 2022) so that any accidental environmental noise could be reduced. The sensors were powered and heated throughout the experiment. We took care of their cleanliness. Ten measurements were collected for each sample. This gives a total of 80 records to be analyzed.

## 5. Results of Measurements

### 5.1. Sensor Response

**Figure 6:**
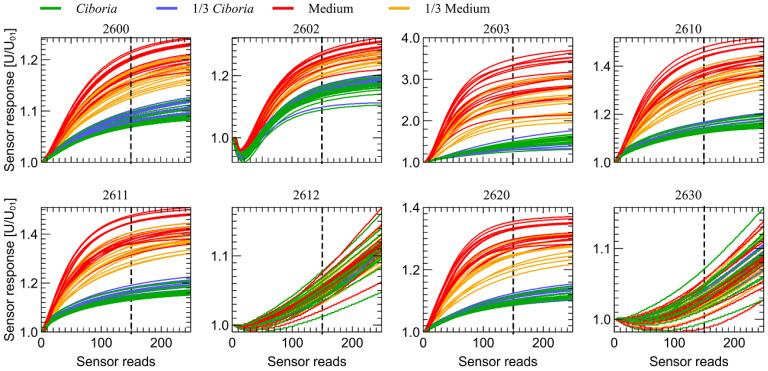
Sensors transient response in the first stage of the observation time (after the opening of the sensor chamber shutter), versus time elapsed from the chamber shutter opening. The sensor’s response voltage is normalized by the voltage at the beginning of the stage of observation time U_01_. Variants of the measured samples are marked by color lines. The sensor type is indicated above the subfigures. The vertical dotted line indicates the observation moment at which data presented in Figure 8 are collected.

In Figure 6, we present the comparison of transient sensor responses collected in the first observation stage when the change of conditions is caused by shutter opening and sensors exposure to the measured gas. As one can notice there is a distinct separation between the data collected during measurements of samples of medium and samples containing *Cyboria batschiana*. Some differences between the results of measurements of samples of fully-covered and partially-covered Petri dishes can be observed, meaning that the concentration of odors influences the captured response. However, the like samples are closely grouped. We can also notice that all used sensors except TGS 2612 and TGS 2630 allow us to detect the presence of fungi odor, which was already found in our previous research [34], in which only the sensor heater temperature modulation mode was explored.

What is encouraging is that we can observe that the results are reproducible and that, especially for *Ciboria* samples, their variability is relatively small. Moreover, the difference between the categories of fully and partially covered by fungi Petri dishes are located very closely.

**Figure 7:**
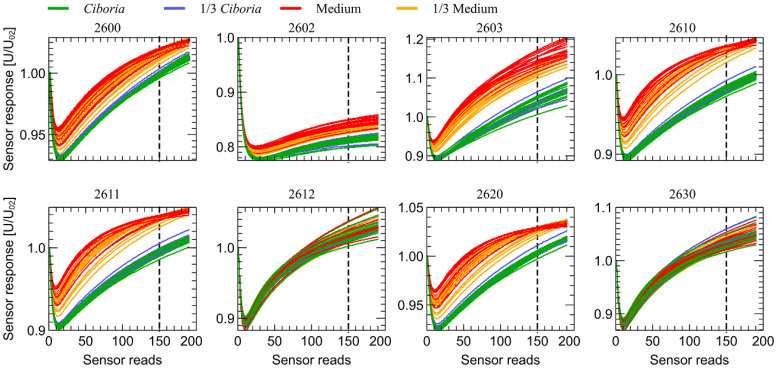
Sensors transient response in the second stage of the observation time (after reduction of the sensor heater voltage), versus time elapsed from the moment of the voltage reduction. The sensor’s response voltage is normalized by the voltage at the beginning of the stage of observation time U_02_. Variants of the measured samples are marked by color lines. The sensor type is indicated above the subfigures. The vertical dotted line indicates the observation moment at which data presented in Figure 9 is collected.

Qualitatively similar results were obtained for the second stage of observation when the sensor transient response was caused by the drop of the sensor’s heater voltage. Such data are presented in Figure 7. Moreover, in that case, the separation between clean and infected samples and the separation between fully and partially covered samples can be noticed. What is important to notice is that the data collected in the second stage of observation are collected when the sensors reached a stationary state after exposure to the measured gas. They are less prone to some initial conditions of non-repeatable shutter opening or potential impurities in cleaning the air before the measurement.

As one can notice in Figure 6, the strongest response to the studied odors is observed for the sensor TGS 2603, for which the measured voltage changed even more than threefold. That may be explained by the target odors of this sensor, presented in Table A1 in Appendix A, which include the odors of waste materials as spoiled foods. We have not performed the analysis of chemical components present in the measured samples, but we can expect that they may be of such a kind. What is also interesting is that the TGS 2603 sensor exhibits the strongest response to measured gases in the temperature modulation mode, as it is presented in Figure 7, in the range from −10% to +20%. In the previous research [32,33,49], we observed that the sensors TGS 2603 and TGS 2602 can efficiently provide the data allowing the detection and differentiation between fungi species.

The results of the third and fourth stages of observation also exhibit very similar features and we present them in the Appendix B.

The collected sensors’ responses during the measurements were reproducible and the baseline had not exhibited any trend indicating the sensor’s poisoning. Supplementary results are presented in Appendix C.

### 5.2. Classification of Measured Samples

Different patterns of the sensor’s response caused by the presence of various odors can be used for classification algorithms and automatic detection of the presence of pathogenic fungi, which is the ultimate goal of the reported research. As we presented in Figure 6 and Figure 7, such patterns can be noticed. However, for a more automatic process, a common practice is to select a limited number of features describing the response curves and use them as input for machine learning classification algorithms.

The most basic feature that can be used for classification is the final steady-state value of the response curve after the sensors are exposed to the studied odor. Other features commonly used in the context of patterns extracted from electronic nose data include basic statistics calculated from the sensor response such as the average value, standard deviation, skewness, and kurtosis [50,51]. In some research, sensor response values at defined or characteristic moments such as the time to reach maximum/minimum of the curve derivative or time to reach, for example, 10%, 25%, or 50% of the sensor response range, or at defined moments such as, e.g., at 1 s, 2 s, etc. [52]. To remove the measurement noise, they are usually evaluated after smoothing the response curve. The sensor response curve can also be fitted by the analytical functions such as polynomial, sigmoid, or exponential, and the fitting parameters are used as the modeling features [53,54]. Yan et al. [50] reviewed the applications of various feature extraction methods in the odor detection domain using an electronic nose. In our previous studies [51], we described an extensive number of features extracted from the sensor response curves, and the selection procedure of modeling features. That approach we followed in other studies [32,33,49]. For the sensor response obtained in the mode of operation of rectangular change of the heater voltage, in the previous work [34], we proposed a list of classification features. Other authors propose such a case different approach [55,56,57].

**Figure 8:**
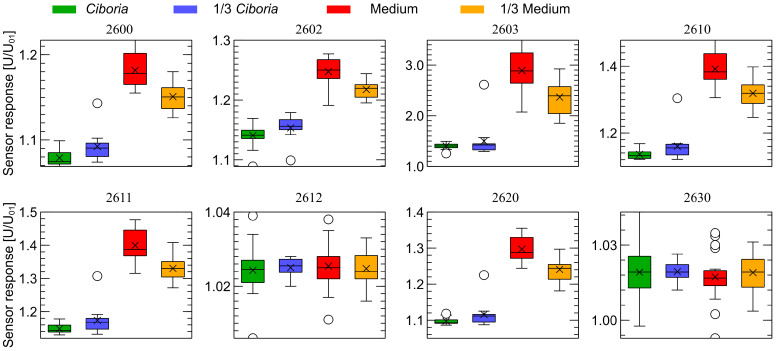
Distribution of the sensor response magnitude collected at the moment of 150 sensor reads from the beginning of the first observation stage (Figure 6). Comparison of four categories of samples used in the experiments.

**Figure 9:**
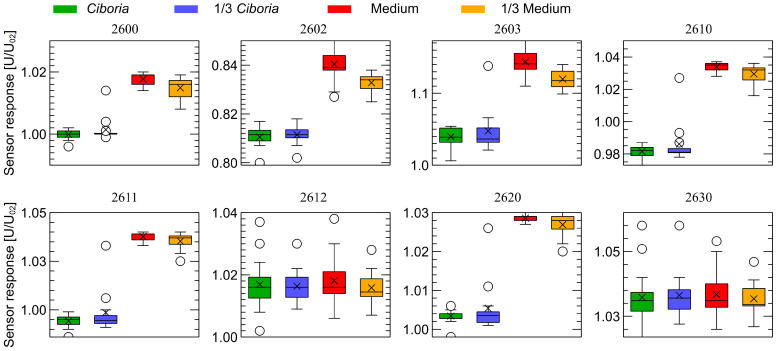
Distribution of the sensor response magnitude collected at the moment of 150 sensor reads from the beginning of the second observation stage (Figure 7). Comparison of four categories of samples used in the experiments.

The patterns observed in Figure 6 and Figure 7 are very distinctive. For visualization purposes, to demonstrate that even a single, very simple feature extracted from the response of each sensor can be used for classification, we decided to extract just a sensor response magnitude at 150 sensor’s read, counted from the beginning of each observation stage (1 min 53 s). We present such data in Figure 8 and Figure 9 for the first and second observation stages, respectively.

As one can notice, in Figure 8 and Figure 9, there is a clear separation between healthy and infected samples. In our preliminary studies, we are interested only in the demonstration of the possibility of detection of the odors of *Ciboria* infection; thus, we have not tested the possibility to build classification models allowing us to estimate the odor intensity, which in our case would be the differentiation between of fully covered and partially covered Petri dishes. For differentiation between healthy and infected categories, the data collected by many of the sensors, as presented in Figure 8 and Figure 9, do not overlap at all and thus allow us to perfectly differentiate between these two groups of samples. (There is only one outlier record, which one can notice.) Having a such clear separation of data, we have not followed the analysis in training the machine learning models, as in our opinion, the results would not be interesting.

## 6. Discussion

### 6.1. Detection of Pathogenic Infections of Acorns

The utility of the device in forest protection practice cannot be overstated, as it makes the work of nursery foresters easier and more efficient. Especially in the spring, they struggle with seeding different seed varieties and must decide whether a lot is suitable for seeding. If the decision to use the inferior seed turns out to be wrong, the emergence achieved may not outweigh the cost of soil preparation and seeding. In addition, the planting seed infected with pathogens risks contaminating the soil for many years. If the introduced pathogen survives, e.g., on dead plant debris, it will also infect and damage healthy seeds at the next sowing, which will also negatively affect the production of healthy seedlings. Therefore, it is necessary to develop methods for the early detection of pathogens that are already in the seed or seedlings to prevent the pathogens from spreading further into the forest. Frequently, *Ciboria batschiana* becomes established in oak stands and is transmitted to nurseries. If detected there in time, it can be eradicated with available chemicals or by thermotherapy. The latter method, which consists of a two-hour seed bath at 41 °C, is very effective as long as secondary infections do not occur during seed storage. Although seeds are additionally treated with fungicides to protect them from other fungi, the natural resistance of fungi to pesticides is observed after years of use. Chemicals are also not environmentally safe, especially when they enter groundwater. For this reason, the European Commission has decided to restrict their use (Directive on Integrated Pest Management). The search for new methods of early detection of pathogens already in nurseries is in line with the policy of the European Union and deserves to be spread.

### 6.2. Advantages and Applications of the Open Sensor Chamber

There are several applications of odor measurements by electronic noses, for which, in our opinion, the proposed open sensor chamber may have some advantages. The electronic noses construction, in which measured gas is transported to the sensor chamber, may require substantial volumes of gas samples. The gas flow needs to be maintained, sometimes even in scales of minutes, as may be a characteristic time of sensor response. During that time, one needs to maintain a stable composition of the measured odor. Moreover, in some of the presented constructions of electronic noses, a pneumatic system used for gas delivery to the sensor’s area may lead to the dilution of the gas samples by neutral carrier gas, usually clean air. In various applications, gas samples may be obtained substantially without any problems. This is, for example, the case of environmental monitoring. There are also cases when the measured odors are in heavy concentration, and the dilution of it also does not harm detection performance by the electronic noses.

There are some applications of odor detection in which the volume of the available gas sample is relatively small, for example, in a range of a few cubic centimeters, and one cannot achieve a flow of a gas through a sensor chamber for a minute or so, without gas dilution. Such is the case with the biological sample presented in our experiment. A similar case that one can notice in other reports is a medical application of monitoring the state of wounds [58,59,60]. In such applications, an electronic nose should not be used directly in the world environment, as such an instrument may be difficult to sterilize and may be a source of contamination. Samples of dressing may be then used, and the volume of gas available for measurements is small.

The proposed construction of a sensor chamber allows placing sensors very close to the measured sample, achieving only a small degree of odor dilution and maintaining the stable composition of analyzed gas during the required time of measurement. A disadvantage of previous constructions of electronic noses designed by our team [32,33,34], and most electronic noses reviewed in Section 2.2 consists of difficulties in the capture of the baseline signal when sensors must be placed in reference gas, which is usually clean air. The design of a sensor chamber with a shutter and simple pneumatic system used only to transport clean air allows us to overcome some drawbacks of such constructions and still keep a very low cost of electronic nose devices.

Another approach for electronic nose measurements of small volumes of odors or odors of weak concentration may be an application of pre-concentration units [61,62,63,64,65,66,67].

## 7. Summary and Conclusions

A low-cost electronic nose sensor’s gas chamber design is proposed, in which a measured sample of the gas is not transported to the sensors’ area. A simple pneumatic system is used to provide clean air to determine the sensor’s baseline response level and sensor cleaning.

The proposed sensor chamber combines the advantages of two approaches to the construction of electronic noses (i) devices with open sensor chambers, when the sensors are exposed directly to the measured gas environment, (ii) closed sensor chambers when the measured gas is transported into the chamber by a pneumatic system. As in the first case, the measurement does not require a dilution of the sample by a carrier gas, which is a common issue in the second case. The advantage similar to the second type of chamber construction is the possibility of efficiently cleaning sensors between the measurement cycles of various samples. The proposed chamber is well suited to measurements of small volumes of gases, with good detection sensitivity.

It has been demonstrated that the proposed setup allows efficient cleaning of the sensors during the measurement cycles and relaxation of the sensor response to the previous state, close to that observed at the beginning of the measurement cycle, before the sensor’s exposure to the studied odor. It has been observed that the baseline level is maintained during several hours of repetitive measurement cycles, without the exhibition of symptoms of sensor poisoning.

In the electronic nose, we used eight metal oxide sensors of the TGS series of Figaro Co. (Japan). The used electrical circuit of allows modulation the sensors’ heater voltage, which leads to the modulation of the operation temperature of the sensors.

The proposed electronic nose device was applied to differentiate between odors emitted by healthy medium samples and infected samples of *Ciboria batschiana*; oak acorns pathogenic fungi.

The following experimental variants were used: (i) samples fully covered by the fungus, (ii) medium, (iii) samples partially covered by the fungus, and (iv) medium.

It has been demonstrated that the transient signals of the sensor’s response collected in four stages of the measurement process exhibit distinct patterns, allowing for the differentiation between studied sample categories. (i) The response to the change from clean air to measured gas conditions. (ii) The response to a rectangular drop of the sensor’s heater voltage. (iii) The response to a rectangular increase in the sensor’s heater voltage. (iv) The relaxation after changing the sensor’s condition from the measured gas to clean air.

We have found a distinct separation between the data collected during measurements of samples of medium and samples containing *Cyboria batschiana*. Some differences between the results of the measurements of samples of fully-covered and partially-covered Petri dishes can be observed, meaning that the concentration of odors influences the captured response. However, the like samples are closely grouped. For the visualization purpose, we extracted from the sensor response curves the magnitude of the response at the time moment of 150 sensor reads from the beginning of each observation stage. That gave a single feature for each sensor, allowing perfect differentiation between healthy and infected samples.

## 8. Patents

A patent application for the open gas sensor chamber of an electronic nose has been submitted by the authors.

## Figures and Tables

**Figure A1:**
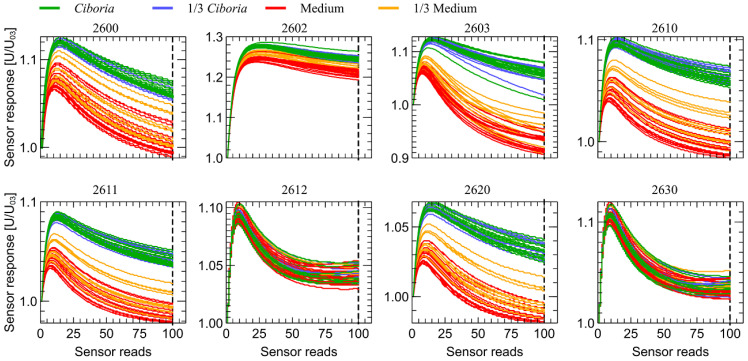
Sensors’ transient response in the third stage of the observation time (after the increase in the sensor heater voltage), versus time elapsed from the moment of voltage increase. The sensor’s response voltage is expressed as normalized by the voltage at the beginning of the stage of observation time U_03_. Variants of the measured samples are marked by color lines. The sensor type is indicated above the subfigures. The vertical dotted line indicates the observation moment at which data presented in Figure A3 is collected.

**Figure A2:**
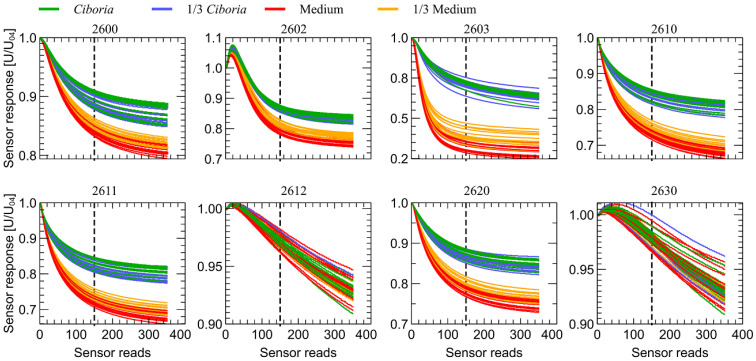
Sensors’ transient response in the fourth stage of the observation time (after the closing of the sensor chamber shutter), versus time elapsed from the moment of voltage reduction. The sensor’s response voltage is expressed as normalized by the voltage at the beginning of the stage of observation time U_04_. Variants of the measured samples are marked by color lines. The sensor type is indicated above the subfigures. The vertical dotted line indicates the observation moment at which data presented in Figure A4 are collected.

**Figure A3:**
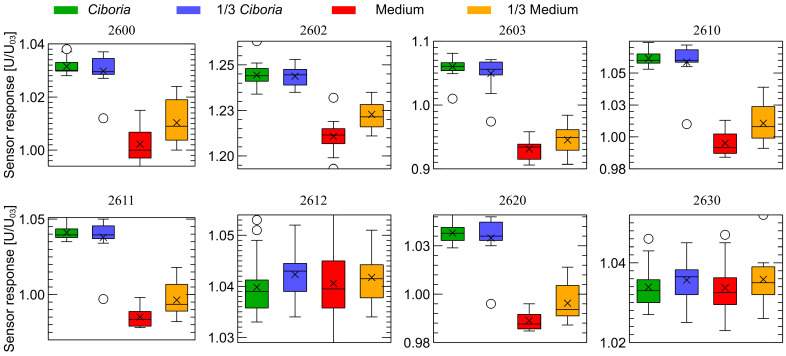
Distribution of the sensor response magnitude collected at the moment of 100 sensor reads from the beginning of the third observation stage (Figure A1). Comparison of four categories of samples used in the experiments.

**Figure A4:**
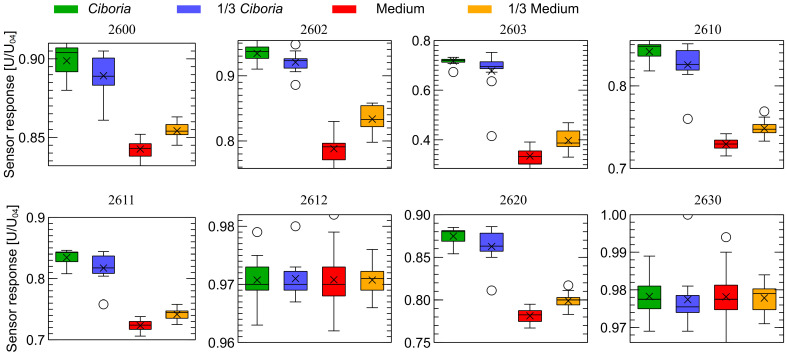
Distribution of the sensor response magnitude collected at the moment of 150 sensor reads from the beginning of the fourth observation stage (Figure A2). Comparison of four categories of samples used in the experiments.

**Figure A5:**
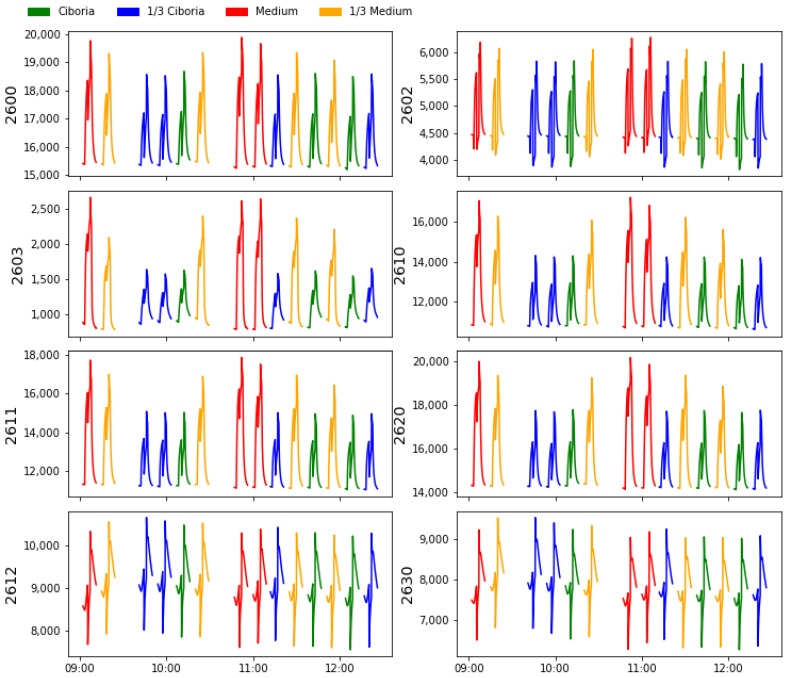
Raw sensor response expressed voltage measured on the resistor (output from analog–digital converter) during one day of the measurement. Hour or the measurement is presented in the *x*-axis.

## Data Availability

Not applicable.

## Appendix A. Electronic Nose Sensors

**Table A1:** List of sensor models used in our electronic nose device and target odors and gases.

Sensor	Target Gas Detection
TGS 2600	Has a high sensitivity to low concentrations of gaseous air contaminants such as hydrogen and carbon monoxide, which exist in cigarette smoke. The sensor can detect hydrogen at a level of several ppm.
TGS 2600	Has a high sensitivity to low concentrations of gaseous air contaminants such as hydrogen and carbon monoxide, which exist in cigarette smoke. The sensor can detect hydrogen at a level of several ppm.
TGS 2602	Has high sensitivity to low concentrations of odorous gases such as ammonia and H_2_S generated from waste materials in office and home environments. The sensor also has a high sensitivity to low concentrations of VOCs, such as toluene emitted from wood finishing and construction products.
TGS 2603	Has high sensitivity to low concentrations of odorous gases such as amine-series and sulfurous odors generated from waste materials or spoiled foods such as fish.
TGS 2610	Uses filter material in its housing, eliminating the influence of interference gases such as alcohol, resulting in a highly selective response to LP gas.
TGS 2611	Uses filter material in its housing which eliminates the influence of interference gases such as alcohol, resulting in a highly selective response to methane gas.
TGS 2612	Has high sensitivity to methane, propane and butane, making it ideal for LNG and LPG monitoring. Due to its low sensitivity to alcohol vapors (a typical interference gas in the residential environment), the sensor is ideal for consumer market gas alarms.
TGS 2620	Has high sensitivity to organic solvents and other volatile vapors, making it suitable for organic vapor detectors/alarms.
TGS 2630	Has high sensitivity to low GWP (Global Warming Potential), low-flammable refrigerant gases such as R-32 and R-1234yf, as well as to R-404a and R-410a, which are commonly used in air conditioning and refrigeration systems. Uses filter material in its housing to eliminate the influence of interference gases such as alcohol, resulting in a highly selective response to low-flammable refrigerant gases.

## Appendix C. Sensor Response during One Day of Measurements

In Figure A5 we present the raw sensor response captured during one day of the measurement. We include this figure as an example of the ability of the constructed device to efficiently clean sensors after their exposition on the measured odor. As one can notice here, for all sensors, the captured signal after the measurement cycle reaches a magnitude close to the initial value before the cycle. The cleaning of sensors is continued after the end of the data collection time during the measurement cycle and we can observe that the signal baseline of the subsequent measurement cycle is close to that of the previous cycle. That confirms that we do not observe significant sensor poisoning during the measurement procedure. That also confirms that the measurement conditions are kept constant and we do not observe significant trends during the measurement day. The measurements were performed in conditions of externally controlled temperature and humidity in a laminar flow chamber (Section 4.2), but also we do not observe other trends that could be associated, for example, with the presence and change of background interfering odors. That is an improvement when we compare it to our previous results, for example, presented in Ref. [32] (Supplementary Materials), where we have not used a dedicated sensor chamber.
